# Classical vs. Retrograde Endoscopic Dacryocystorhinostomy: Analyses and Comparison of the Results

**DOI:** 10.3390/jcm13133824

**Published:** 2024-06-29

**Authors:** Matteo Alicandri-Ciufelli, Daniela Lucidi, Elisa Aggazzotti Cavazza, Paolo Russo, Cinzia Del Giovane, Daniele Marchioni, Federico Calvaruso

**Affiliations:** 1 Department of Otorhinolaryngology Head and Neck Surgery, University Hospital of Modena, 41124 Modena, Italy; matteo.alicandriciufelli@unimore.it (M.A.-C.); dani.lucidi@gmail.com (D.L.); elisa.aggazzotti@libero.it (E.A.C.); daniele.marchioni@unimore.it (D.M.); 2 Otolaryngology Unit, Department of Surgery, Azienda USL-IRCCS di Reggio Emilia, 42123 Reggio Emilia, Italy; paolorusso.orl@gmail.com; 3 Department of Medical and Surgical Sciences for Children and Adults, University-Hospital of Modena and Reggio Emilia, 41124 Modena, Italy; cinzia.delgiovane@unimore.it; 4 Institute of Primary Health Care (BIHAM), University of Bern, 3012 Bern, Switzerland

**Keywords:** dacryocystorhinostomy, endoscopic dacryocystorhinostomy, retrograde dacryocystorhinostomy

## Abstract

**Background:** In endoscopic dacryocystorhinostomy (DCR), surgical landmarks such as the maxillary line (ML) and the axilla of the middle turbinate (MT) guide the surgeon in identifying the lacrimal sac. The primary surgical risk associated with the classical technique, which involves directly opening the lacrimal sac, is the height of the bone drilling on the projection of the lateral wall of the nasal fossa. This poses a significant risk of damaging the orbit, the floor of the frontal sinus, and the anterior skull base. Furthermore, the anatomical variability in size and location of the lacrimal sac poses a risk for difficult and precise surgical identification. Recently, a ‘retrograde’ technique has been introduced to safely identify and expose the lacrimal sac. The aim of this study is to compare the results of retrograde DCR (rDCR) to a classic technique (clDCR), in terms of clinical recurrence and complications. **Methods:** A retrospective study on a cohort of 35 patients who underwent DCR at the ENT Department of the Modena University Hospital between January 2010 and October 2022 (18 clDCR and 17 rDCR) was performed. Minimum postoperative follow-up for inclusion was 12 months. We used the Fisher’s exact test to compare the two techniques, comparing functional outcomes and clinical recurrence rates. **Results:** Clinical recurrence of nasolacrimal stenosis in clDCR patients was 50%, compared to 6% in those who underwent rDCR (*p*-value 0.005). Postoperative surgical complications were not significantly different between the two groups (*p* > 0.05). **Conclusions:** rDCR is a safe technique and has been shown to be a statistically more effective surgical technique than clDCR in reducing clinical recurrence rates.

## 1. Introduction

Endoscopic DCR is the most common surgical procedure for treating NLD obstruction. This has been affirmed since the 1980s in comparison to previous open approaches to the lacrimal sac (LS) [1,2,3]. Since then, several surgical variants of endoscopic DCR have been described to improve outcomes [4,5].

**Figure 1:**
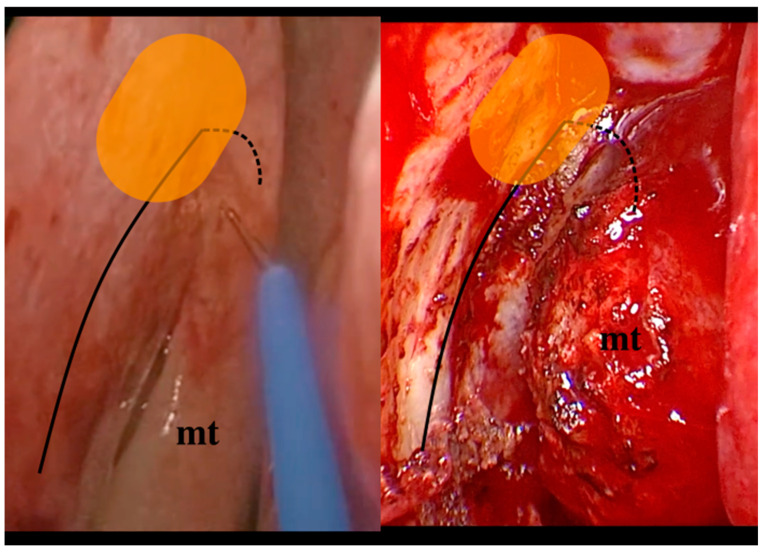
The images demonstrate the surgical landmarks of the clDCR. The continuous line represents the projection of the maxillary line (ML), the dotted line represents the middle turbinate axilla and its insertion on the lateral wall of the nasal cavity, and the orange area represents the hypothesized location of the lacrimal sac. mt: middle turbinate.

The surgical landmarks of the classic DCR technique approaching the lacrimal fossa are represented by the axilla of the middle turbinate (MT) and its insertion on the lateral wall of the nasal cavity and the maxillary line (ML). It is widely acknowledged that the location of the lacrimal sac and the extent of the tear duct in relation to the main surgical landmarks can vary significantly among individuals. It has been amply demonstrated that the localization of the lacrimal sac can vary, not only in the antero-posterior sense and therefore in relation to findings such as ML, but also in the cranio-caudal sense [6,7,8]. The anatomical region at risk is the superior projection of the lacrimal sac, which can exceed the height of the fronto-ethmoidal recess. At this level, surgical complications from closing the frontal ostium or, even worse, from damaging the anterior skull base are uncommon but possible [9,10,11]. In Figure 1, the endoscopic view of the surgical landmarks of cDRC is reported.

In recent years, DCR techniques have changed toward less aggressive approaches in terms of saving as much nasal mucosa as possible. A large bone drilling without an adequate mucous-periosteal flap beforehand can cause significant damage to the nasal mucosa. Proper bone work and the use of a tissue-sparing procedure are crucial for the correct healing of the surgical site.

The aim of the present study is to evaluate the postoperative functional results—in terms of the clinical recurrence rate of nasolacrimal pathology (recurrence of epiphora or dacryocystitis) and postoperative complications of a variant of dacryocystorhinostomy known as retrograde dacryocystorhinostomy (rDCR), described in 2022 [12]—as compared with the classical technique (clDCR). rDCR differs from classical techniques due to elevation of a larger, superiorly based flap, early identification of NLD at the level of the axilla of the inferior turbinate, then following the lacrimal pathway in a retrograde fashion (from inferiorly to superiorly) compared to the lacrimal outflow, progressively drilling over the lacrimal bone to uncover the lacrimal sac. Secondly, a correlation of the surgical outcomes of clDCR and rDCR and selected clinical and anatomical factors (such as allergic or non-allergic chronic rhinitis, turbinate hypertrophy, septal deviation, concha bullosa, and agger nasi pneumatization) was performed.

## 2. Materials and Methods

A retrospective chart review of patients who underwent endoscopic DCR at the Otolaryngology Department of the Modena University Hospital between January 2010 and October 2022 was performed between September and November 2023. Inclusion criteria were as follows: patients who underwent primary endoscopic DCR operated on by a single surgeon (MAC) and minimum follow-up time of 12 months. For all included patients, a detailed assessment of clinical factors, such as the presence of allergic or non-allergic chronic rhinitis and local anatomical factors such as septal deviation, nasal turbinates hypertrophy, concha bullosa, and cellularity of the agger nasi, was retrieved based on clinical data and/or CT scans.

All included patients underwent a preoperative radiological study using either facial CT or Dacrio-CT. The surgical procedure was performed in all cases using a rigid optic with a 4 mm diameter while the patient was under general anesthesia. At the start of the surgical procedure, nasal mucosal decongestion is achieved by using impregnated cottons containing adrenaline, and an infiltration with mepivacaine 1%—epinephrine 1:200,000 is performed at the level of the MT axilla and the maxillary frontal process. The procedure begins with the mucosal incision and preparation of the mucous-periosteal flap. The classic DCR technique consists of the following steps: a vertical incision is made anteriorly to the ML and superiorly to the axilla of the MT and extended downward by roughly 2–3 cm. Secondly, a second vertical incision is made parallel and anteriorly (10–15 mm) to the previous incision. To join the caudal ends of the two vertical incisions, the surgeon makes a horizontal incision. The mucoperiosteal flap is created and stored superiorly at this stage. Subsequently, the surgeon, based on conventional landmarks which are the maxillary line and middle turbinate axilla, finds and drills the frontal process of the maxillary bone and the lacrimal bone, in an anterior-to-posterior direction, opening the lacrimal sac. The opening of the excretion pathway requires a posteriorly hinged flap, made by using a sickle knife. Finally, the mucous-periosteal flap is repositioned.

**Figure 2:**
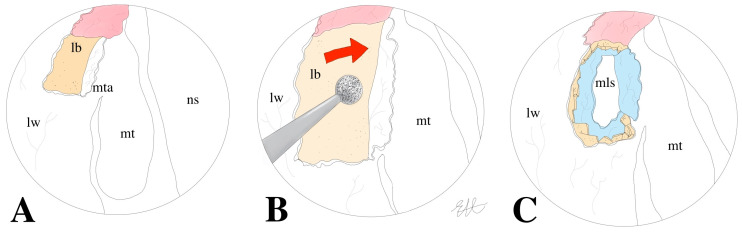
Surgical steps of the clDCR. Panel (**A**): the mucoperiosteal flap is harvested at the level of the middle turbinate axilla. Panel (**B**): the area corresponding to the lacrimal bone is drilled. Panel (**C**): exposure and marsupialization of the lacrimal sac. lw: lateral wall; mta: middle turbinate axilla; mt: middle turbinate; lb: lacrimal bone; ns: nasal septum; mls: marsupialized lacrimal sac.

Figure 2A–C illustrate the localization of the mucoperiosteal flap in the classical technique and the proximity of the bone drilling to the middle turbinate.

**Figure 3:**
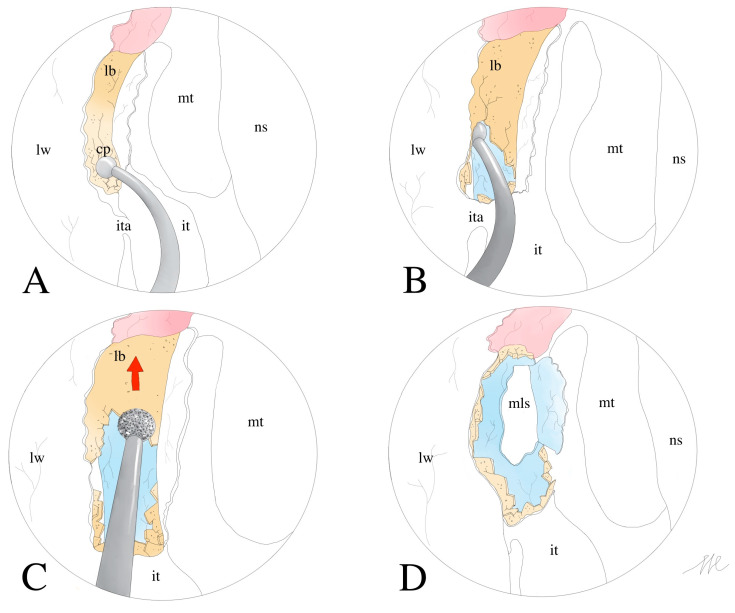
Surgical steps of the rDCR. Panel (**A**): height and width of the mucoperiosteal flap at the axilla of the inferior turbinate are shown. Panel (**B**): identification of the NLD crack point. Panel (**C**): caudo-directional cranial bone drilling until complete identification of the bone and the lacrimal sac. Panel (**D**): marsupialization of the lacrimal sac. lw: lateral wall; ita: inferior turbinate axilla; it: inferior turbinate; cp: crack point of NLD; lb: lacrimal bone; ns: nasal septum; mt: middle turbinate; mls: marsupialized lacrimal sac.

The retrograde technique differs from the classical technique as follows: the horizontal incision of the flap is wider and more caudal than in the classical technique, at the level of the insertion of the inferior turbinate. The nasolacrimal duct (NLD) is safely identified at the level of the inferior turbinate, and caudo-cranial drilling is then performed to ensure correct identification and exposure of the lacrimal sac. Once the wider caudal mucoperiosteal flap has been created, the rDCR surgeon uses a curved dissector to locate and fracture the NLD just above the lateral insertion of the inferior turbinate (Figure 3A). When the thickness and consistency of the bone do not allow for immediate decompression of the NLD, a small-caliber diamond cutter (3–4 mm) can be useful. The surgical dissection should proceed in a caudo-cranial direction (retrograde pathway compared to the lacrimal outflow) along the lacrimal canal (Figure 3B). Then, the bone decompression is extended by a diamond burr to uncover the entire lacrimal canal, always by a caudo-cranial direction (Figure 3C). The lacrimal sac can be finally incised and marsupialized in the same fashion as the clDCR (Figure 3D).

In 2020, the surgeon, who had always performed the classical technique up to that point, chose to change the surgical procedure by performing the retrograde technique in all cases.

In selected cases, a nasolacrimal silicone stenting (BIKA tube) was placed at the end of the procedure, namely in most clDCR and in those rDCR where a fibrotic and scarred lacrimal sac was visualized, and in those cases where a pre-sac component of the obstruction was suspected. Finally, resorbable hemostatic material was placed to control any bleeding. All patients underwent periodic clinical re-evaluation and nasal endoscopy during the postoperative evaluation time. In cases where BIKA stenting was positioned, the removal was performed 2 months after surgery. Recurrence of nasolacrimal stenosis was evaluated clinically as the relapse of symptoms such as epiphora and/or dacryocystitis.

### Statistical Analysis

The data collected from a dedicated Microsoft® Excel® (for Microsoft 365 MSO, version 2404 Build 16.0.17531.20190) dataset underwent a descriptive analysis to provide an assessment of the overall cohort characteristics. Student’s *t*-test was used for continuous variables with normal distribution, while Mann–Whitney U test was adopted for continuous variables without a normal distribution. Comparisons between groups were performed by Pearson’s chi-square or Fisher’s exact test for discrete variables, as appropriate. The association between outcomes of the two techniques was obtained by Pearson’s correlation test. The statistical analysis was performed using STATA 18.1 (StataCorp LP, Texas, USA) for Windows. The results were considered as significant for *p* values < 0.05 with a confidence interval of 95%. For this kind of retrospective investigation, the Institutional Review Board (IRB) of the University Hospital of Modena does not require a formal ethical assessment. This study was performed according to the Declaration of Helsinki.

## 3. Results

Table 1 summarizes the general information of the patient cohort included in the study. A total cohort of 35 patients was identified, 17 (13 women; 4 men) in the rDCR group and 18 (13 women; 5 men) in the clDCR group.

Table 1 summarizes the general information of the patient cohort included in this study. This study included patients with a mean age of 66 years, with no difference in age between the two groups (rDCR: 64; clDCR: 68; *p* = 0.396). In 80% of cases (28/35), the nasolacrimal pathology was unilateral (laterality: 14 right; 14 left), while in the remaining 20% of cases (7/35), the patient had bilateral involvement of the nasolacrimal system. Clinically, the most common symptoms observed were epiphora, present in 77% (27/35) of the included patients, and acute/recurrent dacryocystitis, present in 68.5% (25/35). Enophthalmos was observed in one case (3%). In 17% (6/35) of the patients, previous nasal surgery was observed, mainly septoplasty. Overall, 54% (19/35) of the patients had at least one of the following concomitant clinical and anatomical factors: non-allergic and allergic rhinopathies (5/35–14%), hypertrophy of the inferior turbinate (4/35–11%), ipsilateral septal deviation (14/35–40%), concha bullosa (5/35–14%), and hypercellularity of the agger nasi (1/35–3%). In 43% of cases (15/35), nasolacrimal stenting was placed at the end of the surgical procedure. Of these cases, 4 patients underwent rDCR (24%) and 11 patients underwent clDCR (61%). There was no statistically significant difference identified in the onset of post-surgical clinical relapse between patients who received lacrimal stent placement and those who did not (*p* = 0.179). Nasal packing was performed in 91% of cases using resorbable material. One patient in the rDCR group and two patients in the clDCR group underwent non-resorbable packing. The nasal swabs were removed by the second postoperative day.

The clinical recurrence rate of nasolacrimal stenosis was 50% in the clDCR group and 6% in the rDCR group (*p*-value 0.005; Table 2). No age, clinical-anamnestic, or anatomical factors seemed to be associated with clinical recurrence (see Table 3). Postoperative surgical complications occurred in three cases (8.6% of the overall cohort), and they were all early (<24 h post-operatively). Two patients (2/35–6%), equally distributed between the retrograde and classic DCR groups, experienced self-limiting periorbital oedema. One patient in the clDCR group experienced self-limiting postoperative diplopia without any accompanying extrinsic ocular mobility defects. No statistically significant differences were found in the comparison of postoperative complications between the two groups (*p* > 0.05; Table 4).

## 4. Discussion

DCR is the surgical technique of choice for the treatment of nasolacrimal stenosis characterized by disabling and recurrent symptoms. The rationale of the surgical treatment lies in the decompression of the lacrimal system, the incision of the lacrimal sac and its distal canal, and the marsupialization of the lacrimal walls at the level of the nasal mucosa. Numerous dissection studies on cadavers and, later, some case series have made it possible to characterize the transnasal anatomy of the lacrimal sac and the nasolacrimal canal. Given the proximity of the lacrimal canal to very important sinonasal structures, such as the lamina papiracea (LP) and the frontal sinus, in-depth anatomical knowledge of this system is essential for performing endoscopic DCR [9,10]. In fact, the risk of damaging the LP and the orbital contents, as well as the frontal sinus region, may expose patients to even more serious postoperative clinical complications, such as diplopia, oculomotor defects, and Cerebro-Spinal Fluid leak [11]. The classical DCR technique recognizes the lacrimal sac as the surgical target, and the main surgical landmarks are represented by the axilla of the MT and the ML [12,13]. The latter, which represents the anterior limit of the dissection, is the inferior continuation of the maxillary frontal process and, like all other lacrimal sac limiters, is largely thinned and removed [14,15]. Most authors perform mucosal flaps before drilling over the lacrimal pathway, repositioning the flaps at the end of the operation to avoid excessive scarring. Hence, it is thought that preservation of the nasal mucosa is crucial for the healing process of DCR patients and to minimize the risk of clinical relapse. Finally, although the relationship between surgical landmarks and the location of the lacrimal sac is important, there are numerous anatomic variations among different ethnic groups in terms of position and extent of the lacrimal sac. Moreover, lacrimal bones can have several degrees of thickness, and during surgery, the force and the bone work required to break down these limiting bones of the lacrimal sac will vary accordingly [5,6,7,8]. The critical analysis of these anatomical peculiarities, and the need to perform less and less invasive procedures, has led to the development of several anatomical variants. Endoscopic rDCR, which first identifies the NLD at the level of the insertion of the inferior turbinate, allows the lacrimal sac to be identified with certainty and safety. This is then identified and decompressed “retrogradely” compared to the natural outflow of the lacrimal secretions. This technique allows the surgeon to precisely locate the lacrimal sac without the need for “blind” work [12]. As mentioned, the location of the lacrimal sac is not always easy to identify, despite classical surgical landmarks, and it is located in a dangerous anatomical area. In addition, the mucoperiosteal flap in rDCR, although some millimeters larger than in clDCR, can be easily preserved step by step during surgery. Finally, the final repositioning of the mucosa flap in rDCR allows complete coverage of the surgical defect, thanks to bone drilling that is focused and targeted exclusively on the bony structures over the canal and the lacrimal sac.

The success rates observed in our study for the two groups (cDRC and rDCR patients) were extremely different. In fact, the recurrence rate of nasolacrimal stenosis was significantly higher in the clDCR group compared to the rDCR group (50% versus 6% in the rDCR group). This may be due to the certain identification of the lacrimal duct and sac, the immediate understanding of the whole lacrimal tract anatomy and consequent better perception of the cranial limit of the opening, and limited drilling to the bone medial to the lacrimal duct with consequent reduction in scarring.

It is noticeable that the clinical recurrence rate of patients undergoing clDRC is high. However, this is in agreement with some authors. For example, Mohamed SH et al. reported that clDRC had a success rate ranging from 57 to 89 percent [16,17]. Furthermore, the rate of clinical recurrence increases with the duration of follow-up, as reported by Allon R et al. [18]. However, other studies have reported higher effectiveness rates (ranging from 68.8% to 93.1%) for clDCR, which may vary depending on whether the patient is treatment-naive or has undergone previous surgery [19,20,21].

Finally, considering the complication rates, there were no significant differences in postoperative complications between the two groups, contrary to initial assumptions. It is important to note that although all cases of postoperative complications were mild and self-limiting, only one case showed diplopia due to intraconical material irritation (in a clDCR patient). Besides those data, none of the other local and clinical anatomical factors analyzed (septal deviation, turbinate hypertrophy, bullous concha, and agger nasi) were associated with postoperative recurrence. A possible limitation of this study may be the size of the sample analyzed. Therefore, further studies, possibly multicentric, are needed to clarify the outcomes of retrograde DCR on larger cohorts.

## 5. Conclusions

The rDCR is a safe technique used to identify the lacrimal sac. Based on our experience, rDCR has been statistically proven to be a more effective surgical technique than clDCR in reducing postoperative clinical recurrence rates. The principal limitations of this study are the retrospective analysis and the size of the cohort. Furthermore, it is crucial to acknowledge that the enhanced surgical expertise of the first surgeon, which resulted in a greater number of cases being treated using rDCR, could have influenced our findings. Although we believe that the rDCR technique is a simpler and more secure approach to NLD stenosis treatment, it is important to recognize that the experience factor cannot be overlooked. In order to gain a more comprehensive understanding of the discrepancy in clinical outcomes between clDCR and rDCR, prospective and multicentric research is required.

## Figures and Tables

**Table 1:** Description of participants.

	rDCR	clDCR
Patients	17	18
Sex	12 F; 5 M	14 F; 4 M
Mean Age	64	68
Laterality	7 R; 6 L; 4 B	7 R; 8 L; 3 B
Previous Surgery	5	3
Local factors:		
Chronic Nasal Inflammation	3	2
Turbinates Hypertrophy	2	2
Septal Deviation	6	8
Concha Bullosa	3	2
Hypercellularity of Agger Nasi	0	1
Lacrimal Stenting	4	11

**Table 2:** Clinical recurrence and relationship with surgical technique; statistically significant *p*-value in the group of patients who underwent rDCR.

	clDCR n (%)	rDCR n (%)	*p*-Value	Total
Clinical Recurrence				
Yes	9 (50%)	1 (6%)	0.005	10 (29%)
Not	9 (50%)	16 (54%)		25 (71%)

**Table 3:** Relationship between medical history and local clinical factors with functional outcome (clinical relapse).

	Clinical Recurrence		
	Yes	Not	*p*-Value
**Age at Surgery**			0.396
<50	1 (14%)	6 (86%)	
51–65	0 (0%)	4 (100%)	
66–80	6 (33%)	12 (67%)	
>80	3 (50%)	3 (50%)	
**Sex**			0.694
F	7 (27%)	19 (73%)	
M	3 (33%)	6 (67%)	
**Previous Surgery**			0.564
Yes	2 (33%)	4 (67%)	
Not	8 (28%)	21 (72%)	
**Chronic Nasal Inflammation**			0.164
Yes	0 (0%)	5 (100%)	
Not	10 (33%)	20 (67%)	
**Turbinates Hypertrophy**			0.242
Yes	0 (0%)	4 (100%)	
Not	10 (33%)	21 (67%)	
**Septal Deviation**			0.125
Yes	2 (14%)	12 (86%)	
Not	8 (38%)	13 (62%)	
**Concha Bullosa**			0.553
Yes	1 (20%)	4 (80%)	
Not	9 (30%)	21 (70%)	
**Hypercellularity of Agger Nasi**			0.714
Yes	0 (0%)	1 (100%)	
Not	10 (29%)	24 (71%)	
**Lacrimal Stenting**			0.179
Yes	6 (40%)	9 (60%)	
Not	4 (20%)	16 (80%)	

**Table 4:** There was no statistically significant association found between the occurrence of postoperative complications and the surgical technique used.

	clDCR n (%)	rDCR n (%)	*p*-Value	Total
Postoperative Complications				
Yes	2 (11%)	1 (6%)	0.522	3 (8.57%)
Not	16 (89%)	16 (94%)		32 (91.43%)

## Data Availability

The data are unavailable due to considerations related to the protection of individual privacy.
